# A Comparison of the Accuracy of Two Non-dental 3D Printers and a Standard Dental 3D Printer for Printing Dental Models: An In Vitro 3D Analysis

**DOI:** 10.7759/cureus.76339

**Published:** 2024-12-24

**Authors:** Siddharth Chiang, Rami Ammoun, Chih Yen Liu, Awab Abdulmajeed, Sompop Bencharit

**Affiliations:** 1 General Practice, Virginia Commonwealth University School of Dentistry, Richmond, USA; 2 Prosthodontics, Virginia Commonwealth University School of Dentistry, Richmond, USA; 3 Workman School of Dental Medicine, High Point University, High Point, USA

**Keywords:** 3d printer, 3d printing, dental model, digital dentistry, stereolithography

## Abstract

Background: While the majority of dentists and lab techs recommend dental-specific desktop printers, many of them use cheaper, more affordable 3D printers in their practice. The study aimed to compare the accuracy of two commercial non-dental stereolithography (SLA) 3D printers with a dental 3D printer for diagnostic dental casts.

Methods: A prototype stereolithographic (Standard Triangle/Tessellation Language (STL)) model of a dentoform was used as a master model to be printed by three 3D printers (n=10 for each printer). The casts were post-processed according to the individual manufacturer’s recommendation and then scanned using a desktop dental lab scanner to produce STL files. The STL for each cast was superimposed onto the original STL master model file and dimensional discrepancies were measured.

Results: The results demonstrated a clear hierarchy in the accuracy of the 3D-printed models. The dental-specific 3D printer, Form 3B (Formlabs, Somerville, USA), produced models with the highest accuracy, exhibiting an average deviation of 37±5 µm from the original master model. Among the non-dental printers, the Mars 2 Pro (Elegoo, Shenzhen, China) showed reasonable accuracy with an average deviation of 47±22 µm, while the QiDi Tech (Qidi Technology, Zhejiang, China) had a much larger average deviation of 649±75 µm, indicating poor accuracy. Statistical analysis confirmed significant differences in mean deviations between Form 3B and QiDi Tech (t-test; p<0.01) and between Form 3B and Mars 2 Pro (t-test; p=0.05). These findings highlight the superior performance of the dental-specific 3D printer.

Conclusions: Dental-specific desktop SLA 3D printers appear to have better accuracy than non-dental desktop 3D printers. When a non-dental 3D printer is used, the accuracy of these 3D printers may or may not be sufficient for dental applications.

## Introduction

Additive manufacturing, rapid prototyping, or 3D printing technology began with the introduction of stereolithography (SLA) by Chuck Hull in 1984. He introduced the first 3D printer using SLA, known as SLA-1, commercially later in 1987 [1-3]. The technology utilizes the layering process of joining or adding materials with the primary objective of making objects from 3D model data using the layer-by-layer principle. The SLA technology was introduced together with a language specific for 3D printing known as Standard Triangle/Tessellation Language (STL) [1,2]. STL describes a raw, unstructured, triangulated surface by the unit normal and vertices (ordered by the right-hand rule) of the triangles using a 3D Cartesian coordinate system. This original STL format remained relatively unchanged for 22 years until 2009 when an update to the format, dubbed STL 2.0, was released [4].

3D printing technology has been used in dentistry for over two decades, however, the applications of 3D printing technology have been limited in large commercial laboratories until the last few years. Formlabs Form 2 3D printers were one of the first commercially available 3D printing technology used in dentistry [5]. Later, several desktop 3D printers were developed and widely used in dentistry. While 3D printing technology has recently gained popularity over the last decade in dentistry, it comes with two common drawbacks: high cost and limited dental printing options [3]. While dental-specific 3D printers are relatively inexpensive, they still cost more than other commercially available 3D printers that utilize similar SLA technology. For instance, some commercial non-dental 3D printers that utilize similar SLA rapid prototyping technology may cost ~1/10th of the cost of dental-specific 3D printers [6]. It is therefore tempting for clinicians or lab techs to employ a non-dental commercial printer, which may significantly reduce the cost of the fabrication of dental prosthetics or dental casts. In addition, the utilization of non-dental desktop 3D printers may also provide an alternative printing option when the demand for 3D printing skyrockets while there is a shortage of material in the market [7].

3D-printed diagnostic casts have become widely used in dentistry [8]. In addition, 3D-printed diagnostic casts have become popular in the field of orthodontics [9-11]. While 3D-printed diagnostic casts are readily available, efficient, and cost-effective, their accuracy is controversial [12]. Various 3D printing technologies are evolving and becoming better day by day. A recent study suggested that SLA-printed dental casts may be clinically acceptable for a three-unit fixed partial denture [13,14] and a larger or even a full-arch prosthesis [15]. Regardless, some suggest that the utilization of 3D printing technology will soon replace traditional gypsum-based dental cast fabrication [16].

There is an increase in the availability of affordable non-dental 3D printers in the market. Therefore, this study aimed to compare the differences in the accuracy of two commercial non-dental SLA 3D printers, Qidi Tech Shadow 6.0 Pro (Qidi Technology, Zhejiang, China) and Elegoo Mars 2 Pro Mono SLA 3D Printer (Elegoo, Shenzhen, China), and a control SLA dental 3D printer Form 3B (Formlabs, Somerville, USA) for printing diagnostic dental casts. The null hypothesis was that there would be no difference in the accuracy of 3D-printed casts printed using the Qidi Tech and Mars 2 Pro printers and the dental casts printed using the Form 3B printer.

## Materials and methods

**Figure 1:**
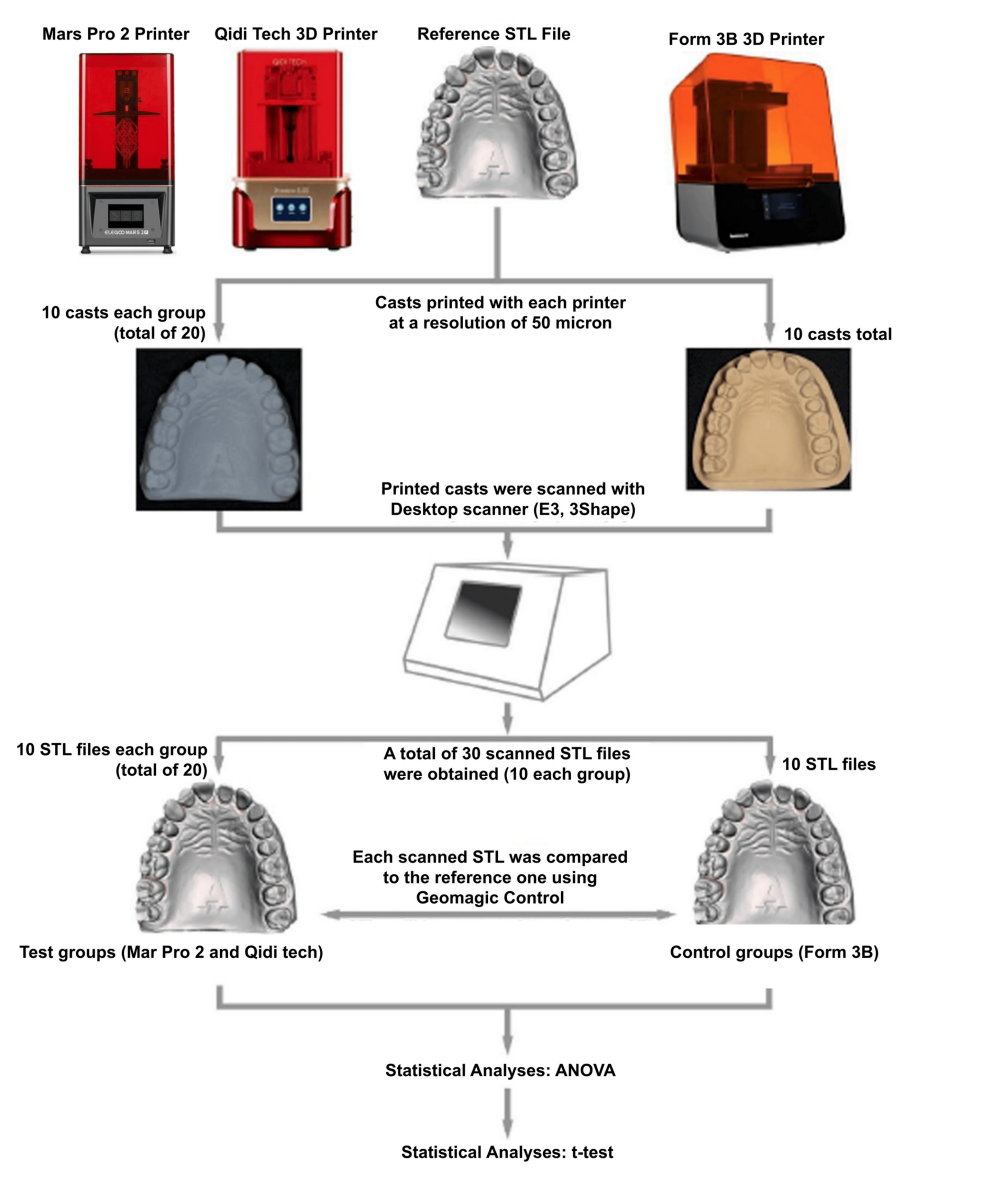
Study design and concept STL: Standard Triangle/Tessellation Language; ANOVA: Analysis of variance

An STL of a diagnostic model, obtained from a scan of a fixed prosthodontic cast for a pre-doctoral training course, was used as a reference master model to be printed by the three selected 3D printers for this study (Figure 1). The Form 3B printer had a printing resolution capacity of 25 to 150 microns, while the Qidi Tech and Mars 2 Pro printers both claimed a printing resolution capacity of 50 microns [17]. A previous study suggested that a printing resolution of 50 micrometers is the optimum layer thickness for printing accuracy using SLA technology, hence 50 microns was the chosen resolution for this study [18]. Ten diagnostic casts were printed for each 3D printer. The model resins used were Dental Model Resin (Formlabs, Somerville, USA) for Form 3B and Grey Rapid Standard Resin (Elegoo, Shenzhen, China) for Qidi Tech as well as Mars 2 Pro. Each specimen was post-processed based on the individual manufacturer’s recommendation.

The sample size for this study was determined based on the primary objective of comparing the dimensional accuracy of 3D-printed diagnostic dental casts produced by two non-dental SLA 3D printers and a dental-specific SLA 3D printer. Preliminary data suggested that the mean dimensional deviation for the dental-specific 3D printer (Form 3B) was approximately 37 µm (SD=5 µm), while the non-dental printers demonstrated larger deviations (e.g., Qidi Tech: mean=649 µm, SD=75 µm). To detect a statistically significant difference in mean deviations between Form 3B and non-dental printers, with a two-tailed significance level of 0.05 and a power of 80%, a minimum of 10 samples per group was calculated as sufficient. This calculation assumed a large effect size (Cohen's d>1.0), given the substantial differences observed in preliminary measurements, and ensured adequate statistical power to identify meaningful differences in dimensional accuracy between the groups.

Each printed diagnostic cast was then scanned using a desktop dental lab scanner (E3, 3Shape, Copenhagen, Denmark) to generate an STL file for each specimen. Each specimen STL file was then superimposed onto the master original reference STL file using best-fit alignment (Geomagic Control X; Geomagnic, Morrisville, USA) similar to previous studies [19-21]. The test STL files were aligned to the reference STL file using the overall best fitting algorithm and 3D analysis was performed using Geomagic Control X. Deviation from the original STL file was reported as average positive and negative values. The average absolute discrepancy (AAD) and maximum absolute discrepancy (MAD) of each STL were used to calculate the average fit of these STL scans. A one-way analysis of variance (ANOVA) with a significance level of 0.05 was used to analyze the AAD and MAD results.

## Results

**Figure 2:**
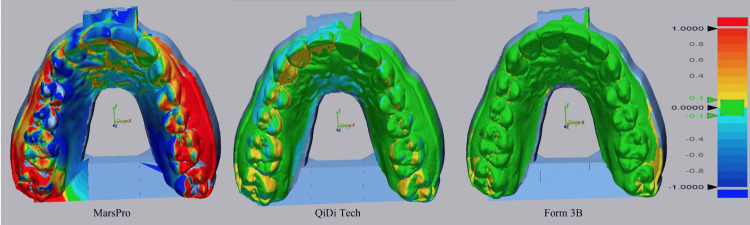
3D comparison of all test groups with original STL file STL: Standard Triangle/Tessellation Language

The comparison of the 3D-printed models produced by Qidi Tech, Mars 2 Pro, and Form 3B printers to the original master reference STL file revealed distinct differences in accuracy and precision among the three printers (Figure 2).

For the Qidi Tech printer, the superimposition analysis with the original master reference STL file showed a mean difference of 649±75 µm, with maximum and minimum differences of 737 µm and 534 µm, respectively. The MAD recorded for the Qidi Tech printer was 3.6 mm (approximately 3600 µm). This indicates that the Qidi Tech printer had the largest deviation from the reference model, suggesting less accuracy and precision in its 3D printing capabilities.

In contrast, the Mars 2 Pro printer produced models with significantly smaller deviations when compared to the original master reference STL file. The mean difference was 47±22 µm, with maximum and minimum differences of 84 µm and 27 µm, respectively. The MAD for the Mars 2 Pro printer was 210 µm. These results indicate that the Mars 2 Pro printer achieved good accuracy, although there was some variability in precision as reflected by the relatively higher standard deviation.

The Form 3B printer demonstrated the highest accuracy among the three printers tested. The superimposition analysis showed a mean difference of 37±5 µm from the original master reference STL file, with maximum and minimum differences of 49 µm and 32 µm, respectively. The MAD for Form 3B was 200 µm, indicating both high accuracy and precision in its 3D-printed models.

**Figure 3:**
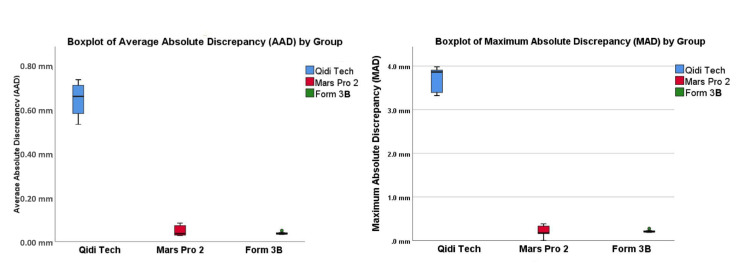
Boxplot of average and maximum absolute discrepancy (AAD and MAD) of each group

Boxplot analysis illustrated the maximum and average absolute discrepancy values for each group (Figure 3). The Qidi Tech printer exhibited the highest values, indicating the least optimal accuracy and precision. The Mars 2 Pro printer displayed good accuracy but showed slight variability in precision. On the other hand, the Form 3B printer yielded the best results in both accuracy and precision, with the lowest discrepancy values.

Statistical analyses using one-way ANOVA (p<0.01) and t-tests confirmed these observations. The mean difference between the Qidi Tech and Form 3B printers was statistically significant (t-test; p<0.01), indicating a substantial difference in performance. However, the difference between the Mars 2 Pro and Form 3B printers was not statistically significant (t-test; p=0.05), suggesting that while Form 3B had a slight edge, both printers performed comparably well in terms of accuracy and precision.

The detailed analyses of AAD and MAD statistics are presented in Table 1. The table provides a comprehensive visual representation of the performance differences among the Qidi Tech, Mars 2 Pro, and Form 3B printers, further emphasizing the superior accuracy and precision of the Form 3B printer.

**Table 1:** The average and maximum absolute discrepancy (AAD and MAD) values

	3D Printer	N	Mean	Standard Deviation	Standard Error	95% Confidence Interval for Mean			
						Lower Bound	Upper Bound	Minimum	Maximum
Average absolute discrepancy (AAD)	Qidi Tech	10	0.649285	0.075133	0.023759	0.595538	0.703032	0.53465	0.7378
	Mars 2 Pro	10	0.047295	0.022552	0.007132	0.031162	0.063428	0.02755	0.0843
	Form 3B	10	0.037355	0.005282	0.00167	0.033577	0.041134	0.03225	0.04955
	Total	30	0.244645	0.294322	0.053736	0.134744	0.354547	0.02755	0.7378
Maximum absolute discrepancy (MAD)	Qidi Tech	10	3.69648	0.277722	0.087823	3.49781	3.89515	3.32295	3.98195
	Mars 2 Pro	10	0.21073	0.117749	0.037236	0.126497	0.294963	0.00165	0.38265
	Form 3B	10	0.20808	0.031133	0.009845	0.185809	0.230351	0.1784	0.272
	Total	30	1.371763	1.680438	0.306805	0.744277	1.999249	0.00165	3.98195

## Discussion

3D printing technology, in the past six to seven years, has become part of routine dental practice. Printing dental casts and models is perhaps one of the most common procedures utilizing this technology. SLA technology is one of the most common 3D printers used in dental office and laboratory settings. While there are many dental dedicated 3D printers commercially available, which may have some manufacturing testing and warranty for dental uses, there are even more 3D printers with similar technology that are not built or tested for dental applications. This study examined three SLA-based 3D printers: Form 3B, which is dedicated to dental applications, and two non-dental 3D printers, Qidi Tech and Mars 2 Pro. The results demonstrated that there were differences in the accuracy and precision values of these 3D printers, and therefore, the null hypothesis was rejected. It is interesting to note that while, as expected, Form 3B’s performance was best in terms of precision and accuracy, one of the non-dental 3D printers, Mars 2 Pro, appeared to have similar accuracy with slightly less precision compared to Form 3B. Qidi Tech demonstrated a clear lack of both accuracy and precision. This suggests that not all 3D printers are created equal. When clinicians or laboratory techs decide to use a non-dental 3D printer, they will have to test the printing accuracy and precision and examine if a particular 3D printer has sufficient accuracy and precision capabilities for dental purposes.

Recent studies have demonstrated the applications of 3D printing for dental casts. Park et al. 2021 compared four 3D printers utilizing fused deposition modeling (FDM), digital light processing (DLP), photopolymer jetting (polyjet), and SLA [12]. In this study, DLP 3D printers demonstrated the best performance in terms of accuracy and precision. The Form 2 (Formlabs, Somerville, USA) printer used in this study provided a similar accuracy of ~53 µm on average discrepancy (similar to 49 µm in this current study). Ellakany et al. 2022 showed that digital casts printed using an SLA 3D printer (Dental Wings, ​​Saxony, Germany) had similar accuracy with conventional stone casts [22]. Digital 3D printed casts demonstrated slightly less accuracy compared to conventional stone casts but the inaccuracies were within the clinically acceptable limits [22,23].

There are many factors that can influence the accuracy and precision of printed dental casts. First, different 3D printing and technology would produce different cast accuracy and precision. Second, the printing layer width; the smaller the layer, the more precise and accurate the printed objects would be [24]. Third, the object orientation may have some effects on the SLA accuracy in printing [20,25,26]. Designs of the cast base can also have some effects on printing accuracy [27]. And finally, different post-processing methods can potentially affect the accuracy of printed casts [19].

This study has some limitations. First, it examined one dental 3D printer and two non-dental 3D printers using SLA technology. There are many more 3D printers in both dental and non-dental sectors. There are also numerous other technologies that may or may not be similar to the SLA technology presented here. Second, the overall discrepancy between the specimen cast and the master reference cast was used here. This only applies to diagnostic casts. For fixed prosthodontics or implantology, marginal accuracy and certain dimensions of the die or between implant analogs may be important. Further studies or analyses will be needed. Third, a pre-made maxillary master cast was used in this study. There may be additional errors and inaccuracies acquired through in vivo processes such as intraoral scanning or impression. Finally, only one setting in printing and post-processing was used. Various printing parameters or post-processing methods may give different results.

## Conclusions

The Form 3B printer, specifically designed for dental purposes, exhibited superior performance with the highest accuracy and precision among the tested printers. The Mars 2 Pro printer, although not explicitly designed for dental use, displayed commendable accuracy, closely approaching that of Form 3B but with lower precision. While non-dental 3D printers like Mars 2 Pro can be viable alternatives, their performance must be carefully evaluated before clinical use. In contrast, the Qidi Tech printer showed significant deviations in both accuracy and precision, making it less suitable for dental applications where high fidelity and consistency are crucial. These findings underscore the necessity for clinicians and laboratory techs to critically assess the capabilities of any 3D printer, especially those not specifically designed for dental applications. Factors such as printing technology, layer resolution, object orientation, and post-processing methods play pivotal roles in determining the final quality of 3D-printed dental casts. Additionally, continuous research and comparative studies are needed to keep pace with technological advancements in 3D printing. Future studies should explore a broader range of printers and printing technologies and consider additional clinical applications such as fixed prosthodontics or implantology where marginal accuracy is paramount.
